# All-atom/coarse-grained hybrid predictions of distribution coefficients in SAMPL5

**DOI:** 10.1007/s10822-016-9926-z

**Published:** 2016-07-26

**Authors:** Samuel Genheden, Jonathan W. Essex

**Affiliations:** 1 Department of Chemistry and Molecular Biology, University of Gothenburg, Box 462, SE-405 30 Göteborg, Sweden; 2 School of Chemistry, University of Southampton, Southampton, SO17 1BJ UK

**Keywords:** Distribution coefficients, Multiscaling, Hybrid model, AA/CG, Elba, SAMPL5

## Abstract

We present blind predictions submitted to the SAMPL5 challenge on calculating distribution coefficients. The predictions were based on estimating the solvation free energies in water and cyclohexane of the 53 compounds in the challenge. These free energies were computed using alchemical free energy simulations based on a hybrid all-atom/coarse-grained model. The compounds were treated with the general Amber force field, whereas the solvent molecules were treated with the Elba coarse-grained model. Considering the simplicity of the solvent model and that we approximate the distribution coefficient with the partition coefficient of the neutral species, the predictions are of good accuracy. The correlation coefficient, *R* is 0.64, 82 % of the predictions have the correct sign and the mean absolute deviation is 1.8 log units. This is on a par with or better than the other simulation-based predictions in the challenge. We present an analysis of the deviations to experiments and compare the predictions to another submission that used all-atom solvent.

**Electronic supplementary material:**

The online version of this article (doi:10.1007/s10822-016-9926-z) contains supplementary material, which is available to authorized users.

## Introduction

Simulations with molecular dynamics (MD) or Monte Carlo provide structural and dynamic information of chemical systems at high resolution and thus are essential complements to wet-lab experiments [1, 2]. The usefulness of such simulations is to a large extent determined by the underlying molecular mechanics force fields, and it is therefore essential to quantify the accuracy of the force field. A basic requirement is that the force field should correctly describe the solvation thermodynamics of small molecules, such as amino-acid analogues or drug-fragments. This has been the strategy to benchmark force fields in numerous publications [3–8]. The ability to truly predict solvation free energies has been assessed by several blind challenges under the SAMPL label [9–12]. The previous four challenges have consisted of a set of hydration free energies, whereas the current challenge is the first one to consider the partitioning between two phases, viz. water and cyclohexane [13].

Molecular simulations are not only limited by the accuracy of the force field, but also the timescales that can be reached [14]. An all-atom (AA) force field, describing each atom individually cannot reach the long time-scales relevant for many biochemical applications unless acceleration techniques [15, 16] or special-purpose hardware [17] is employed. A popular solution to reach longer time-scales is coarse-graining (CG), i.e. grouping atoms into pseudo-particles or beads [18, 19]. This reduces the number of particles that need to be simulated and increases the diffusion rate of the molecules. The CG models are inherently less accurate than AA models: especially CG models of proteins and small molecules currently have a limited usefulness [20]. To remedy this, a hybrid all-atom/coarse-grained model was recently developed, where the most essential part of the system, e.g. a protein or a small molecule, is described with an AA model and the rest of the system, e.g. solvent molecules, are described with a CG model [21]. This model has been used to study small molecules and proteins in water and membrane environments [21, 22]. It has also been used to estimate water/hexane and water/octanol partition coefficients [23]. In this paper, we describe the performance of this hybrid model in the SAMPL5 distribution coefficient challenge.

## Methods

### Solvent models

The solvents, water and cyclohexane, were described with the Elba coarse-grained (CG) model [24]. The Elba water model has been described and extensively benchmarked previously [25]. In Elba, a single water molecule is modelled as a point dipole attached to a Lennard-Jones site (see Figure S1), i.e. a Stockmayer model. The cyclohexane model was developed for the SAMPL5 challenge, with a similar approach to the models of hexane and octane described previously [23]. A single cyclohexane molecule is described by three connected, uncharged, Lennard-Jones sites as shown in Figure S1. The beads have the same parameters as the non-polar bead used to describe lipid tails, except that σ and ε are multiplied by a factor of 0.9. This is a similar reduction applied to ring beads in the MARTINI force field [26]. Therefore, σ = 0.41 nm and ε = 3.19 kJ mol^−1^. The bond length and bond force constant are 0.405 nm and 1269 kJ mol^−1^ nm^−2^, respectively. The validation of this model is discussed further in the Supplementary Material.

### Compound setup

The inputs provided by the organisers for LAMMPS were used as a starting point. The general Amber force field [27, 28] and coordinates of the compounds were retained, whereas the all-atom solvent molecules were coarse-grained using in-house scripts; the all-atom water molecules were replaced by Elba water beads which were positioned at the respective oxygen atom, and the cyclohexane molecules were replaced by Elba cyclohexane molecules with beads placed on the first, third and fifth carbon atom. The system was minimized with 1000 steps of steepest descent and equilibrated for 1.2 ns in the NPT ensemble. A multiple timestep integrator was used [21], propagating the CG–CG non-bonded forces with a 6 fs timestep and all other forces with a 2 fs timestep. The CG–CG non-bonded interactions are a combination of a shifted-force dipole–dipole potential and Lennard-Jones potential. The CG beads interact with the atoms through shifted-force charge–dipole and Lennard-Jones potentials [21]. The cut-off was in all cases 12 Å. The atom–atom non-bonded interactions combine a Lennard-Jones potential with a cut-off at 12 Å and particle–particle particle-mesh Ewald [29] with a 12 Å real-space cut-off. SHAKE [30] was used to constrain covalent bonds involving hydrogen atoms in the compounds. The solvent and compound were coupled to two different Langevin thermostats [31] with a 6 ps coupling constant, keeping the temperature fixed at 298 K. The pressure was kept at 1 atm with a weak-coupling algorithm [32] and a 6 ps coupling constant.

### Free energy simulations

The free energy simulations follow to a large extent a previously outlined method [23]. The Gibbs free energy of solvation was estimated using thermodynamic integration (TI) [33], by coupling the system energy, *U* to a parameter λ. At λ = 0, the compound is fully interacting with the solvent, and at λ = 1, it is completely decoupled, i.e. behaves as a gas-phase molecule. *U* is scaled with a fourth-power function $f(\lambda ) = (1 - \lambda )^{4}$ and twenty-five equally spaced values of λ from 0 to 0.96 were simulated, whereas λ = 1 was estimated by linear extrapolation. The integration was carried out using the trapezium rule. One long simulation was carried out and the value of λ was changed step-wise every 4.8 ns and the initial 1.2 ns at each step was discarded as equilibration. The sampling frequency of the energies for TI was 0.6 ps. In some cases, each value of λ was simulated for 3.6 with 1.2 ns discarded as equilibration, further discussed in the text. For the simulations in water, ten independent repeats were initiated by assigning different starting velocities. For the simulations in cyclohexane, only five independent repeats were used.

### Quality analysis

The quality of the predictions was quantified by the mean absolute deviation (MAD), mean signed deviation (MSD), root-mean-squared deviation (RMSD), Pearson’s correlation coefficient (*R*) and the percentage of correctly predicted signs.

Systematic deviations due to the presence of specific chemical groups were analysed using an established procedure [4]. The BEDROC (Boltzmann-enhanced discrimination of receiver-operating characteristic) metric [34] was computed for the different chemical groups as described previously. The checkmol program [35] (version 0.5) was used to identify the chemical groups, and the BEDROC analysis was performed with the CROC python package [36] (version 1.1). The uncertainty of the BEDROC metric was estimated by 500 bootstrap iterations. A Student’s *t*-test was performed on the absolute deviation for the different groups compared to the entire population of absolute errors.

## Results and discussion

We present predictions for the SAMPL5 distribution coefficient challenge. The predictions were produced by computing the solvation free energy, Δ*G*_solv_, in water and cyclohexane, using molecular dynamics employing an inexpensive hybrid all-atom/coarse-grained (AA/CG) model. The solvent was described with the Elba CG model and the compounds with the general Amber force field. We did not attempt to estimate the solvation free energy of each possible protonation state of the compounds, or even the most likely; rather we computed the solvation free energy of the neutral compound in the tautomeric state given by the organizers and thus approximate the distribution coefficient with the partition coefficient

1 $$\log\ D \approx \log\ P = \frac{{\Delta G_{\text{solv}} ({\text{water}}) - \Delta G_{\text{solv}} ({\text{cyclohexane}})}}{2.3RT}$$

where *R* is the gas constant and *T* the absolute temperature. This is motivated by two considerations: (1) the accurate prediction of Δ*G* for multiple tautomers of a compound would probably be prohibitively expensive, and (2) the estimation of the solvation free energy of ionic compounds is challenging with molecular dynamics simulations. The second consideration is especially true with CG models, which generally do not employ long-range electrostatics.

### Submitted predictions

The Δ*G*_solv_ as well as log *D* are listed in Table 1 for the 53 compounds in the challenge. The standard error of the Δ*G*_solv_ estimates is generally good, between 0.02 and 1.0 kJ/mol for the estimates in cyclohexane and 0.06 and 2.1 kJ/mol for the estimates in water. We used five and ten independent repeats for the cyclohexane and water estimates, respectively, which was deemed necessary after computing estimates for all compounds using only two repeats and only 3.6 ns sampling at each value of λ. It would be prohibitively expensive to reduce the standard error further for some of the estimates in water. The larger standard error of the estimates in water stems from the need to decouple electrostatic interactions (charge–dipole) in this phase, whereas the cyclohexane CG model is uncharged. The submitted predictions were based on 4.8 ns sampling at each value of λ, with 1.2 ns discarded as equilibration. To check that the simulations were converged, we also computed free energies for all compounds in both water and cyclohexane with only 3.6 ns sampling. These estimates are given in the Supplementary Material. The solvation free energies in cyclohexane changed by at most 2.5 kJ/mol when increasing sampling by 1.2 ns, but by only 0.3 kJ/mol on average over all compounds. For only three compounds (**63**, **83** and **92**) the estimate of the solvation free energy changes by more than 1 kJ/mol, and therefore, we submitted the predictions based on 4.8 ns sampling. The solvation free energies in water changed by at most 1.8 kJ/mol when increasing the sampling by 1.2 ns, and by 0.3 kJ/mol on average. For only four compounds (**37**, **67**, **83**, and **84**), the free energy changed by more than 1.0 kJ/mol when increasing the sampling, and thus we consider these estimates to be converged and we submitted the predictions based on 4.8 ns sampling.

**Table 1:** Submitted estimates of log *D* as well as solvation free energies in kJ/mol in water and cyclohexane

Compound	$\Delta G_{\text{solv}} ({\text{water}})$		$\Delta G_{\text{solv}} ({\text{cyclohexane}})$		log *D*		log *D* (exp)	
2	−55.2	±0.1	−63.8	±0.1	1.51	±0.02	1.40	±0.30
3	−47.9	±0.1	−55.3	±0.2	1.29	±0.03	1.90	±0.10
4	−55.7	±0.1	−70.5	±0.1	2.60	±0.03	2.20	±0.30
5	−73.7	±0.2	−74.1	±0.1	0.07	±0.04	−0.86	±0.09
6	−55.8	±0.1	−50.5	±0.1	−0.93	±0.03	−1.02	±0.09
7	−57.3	±0.3	−69.3	±0.2	2.11	±0.06	1.40	±0.30
10	−82.9	±0.1	−58.8	±0.2	−4.23	±0.03	−1.70	±0.40
11	−66.9	±0.1	−63.2	±0.1	−0.65	±0.02	−2.96	±0.08
13	−99.8	±0.1	−89.4	±0.1	−1.83	±0.02	−1.50	±0.40
15	−77.4	±0.2	−59.3	±0.1	−3.17	±0.04	−2.20	±0.30
17	−55.7	±0.3	−77.6	±0.1	3.85	±0.06	2.50	±0.30
19	−79.7	±0.1	−76.4	±0.1	−0.58	±0.03	1.20	±0.40
20	−79.5	±1.9	−63.9	±0.2	−2.74	±0.33	1.60	±0.30
21	−49.4	±0.1	−62.2	±0.1	2.26	±0.02	1.20	±0.30
24	−84.8	±0.2	−85.1	±0.2	0.05	±0.04	1.00	±0.40
26	−76.4	±0.5	−52.5	±0.2	−4.19	±0.09	−2.60	±0.10
27	−87.8	±0.1	−57.3	±0.1	−5.36	±0.03	−1.87	±0.07
33	−56.8	±0.2	−73.6	±0.2	2.95	±0.04	1.80	±0.20
37	−67.9	±0.4	−52.3	±0.2	−2.74	±0.08	−1.50	±0.10
42	−98.3	±0.1	−69.9	±0.2	−4.98	±0.03	−1.10	±0.30
44	−76.0	±0.1	−81.3	±0.1	0.93	±0.02	1.00	±0.40
45	−62.4	±0.1	−50.6	±0.1	−2.07	±0.02	−2.10	±0.20
46	−72.3	±0.1	−71.3	±0.1	−0.19	±0.03	0.20	±0.30
47	−65.5	±0.1	−71.0	±0.2	0.96	±0.04	−0.40	±0.30
48	−85.3	±0.1	−80.2	±0.1	−0.89	±0.02	0.90	±0.40
49	−53.5	±0.1	−53.9	±0.0	0.08	±0.02	1.30	±0.10
50	−65.3	±0.1	−59.8	±0.1	−0.96	±0.02	−3.20	±0.60
55	−53.3	±0.1	−46.6	±0.1	−1.17	±0.02	−1.50	±0.10
56	−59.4	±0.1	−60.3	±0.1	0.16	±0.03	−2.50	±0.10
58	−49.9	±0.1	−54.7	±0.1	0.84	±0.02	0.80	±0.10
59	−61.2	±0.1	−43.5	±0.1	−3.12	±0.03	−1.30	±0.30
60	−90.7	±0.1	−56.2	±0.1	−6.05	±0.03	−3.90	±0.20
61	−39.4	±0.2	−51.7	±0.1	2.15	±0.04	−1.45	±0.09
63	−71.2	±0.4	−59.2	±0.1	−2.10	±0.07	−3.00	±0.40
65	−140.5	±0.2	−143.4	±0.2	0.50	±0.04	0.70	±0.20
67	−50.3	±1.0	−59.6	±0.4	1.63	±0.19	−1.30	±0.30
68	−57.1	±0.3	−73.2	±0.2	2.83	±0.07	1.40	±0.30
69	−82.2	±0.2	−81.9	±0.3	−0.06	±0.06	−1.30	±0.30
70	−32.1	±0.1	−62.4	±0.2	5.31	±0.03	1.60	±0.30
71	−68.0	±0.2	−66.3	±0.1	−0.29	±0.04	−0.10	±0.50
72	−32.2	±0.1	−57.1	±0.1	4.36	±0.03	0.60	±0.30
74	−132.8	±0.2	−75.8	±0.2	−10.00	±0.05	−1.90	±0.30
75	−51.6	±0.5	−66.1	±0.3	2.56	±0.11	−2.80	±0.30
80	−71.1	±0.1	−58.9	±0.1	−2.14	±0.02	−2.20	±0.20
81	−80.1	±1.4	−66.5	±0.7	−2.39	±0.28	−2.20	±0.30
82	−37.5	±0.3	−77.0	±0.2	6.94	±0.06	2.50	±0.40
83	−165.1	±1.9	−162.2	±0.6	−0.50	±0.35	−1.90	±0.40
84	−67.4	±0.9	−79.2	±0.6	2.08	±0.19	0.00	±0.20
85	−83.8	±0.1	−60.3	±0.0	−4.12	±0.02	−2.20	±0.40
86	−58.1	±0.6	−84.8	±0.4	4.68	±0.13	0.70	±0.20
88	−64.2	±0.2	−62.1	±0.2	−0.36	±0.05	−1.90	±0.30
90	−53.8	±0.1	−75.4	±0.2	3.78	±0.04	0.80	±0.20
92	−107.3	±2.1	−117.1	±1.0	1.71	±0.41	−0.40	±0.30
MAD					1.81			
MSD					0.31			
RMSD					2.42			
*R*					0.64			

**Fig. 1:**
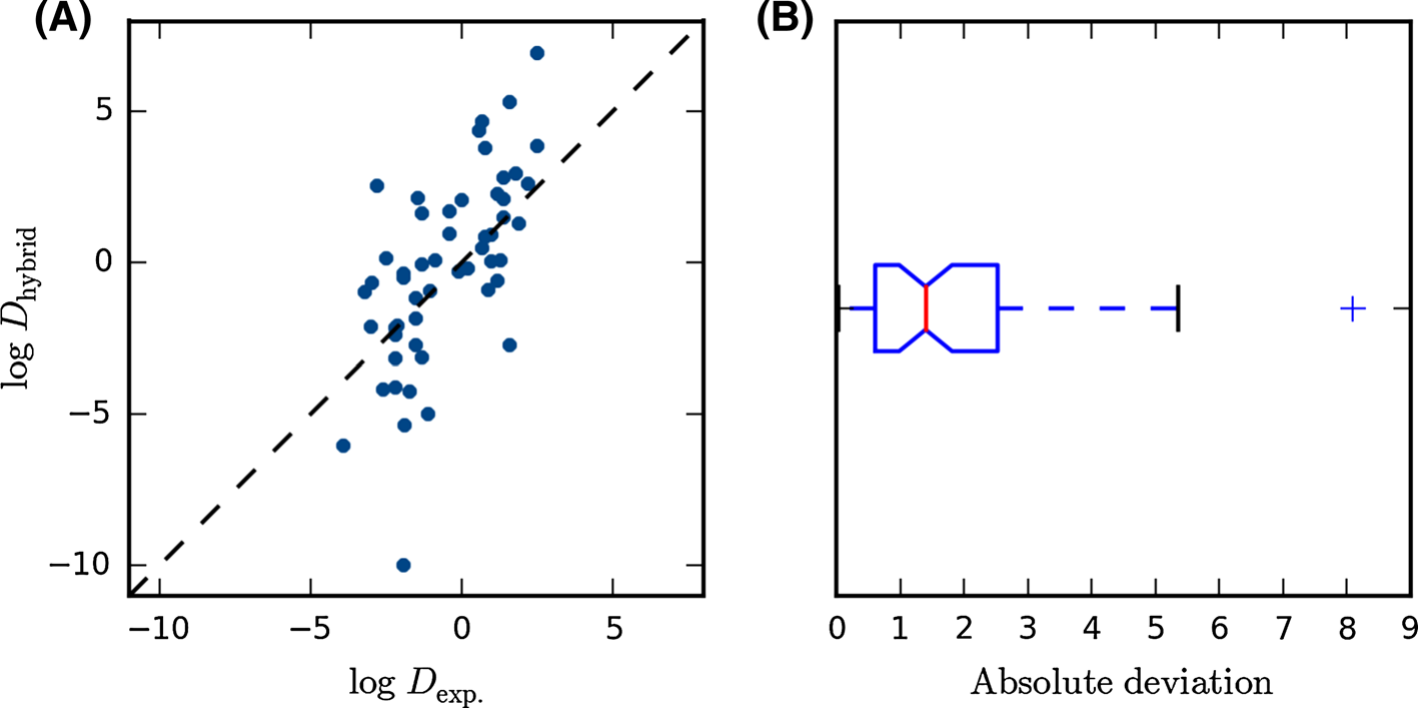
**a** Experimental versus predicted log *D* and **b** *boxplot* of absolute deviations compared to experiments. The *vertical line* in the middle of the box shows the median and the box covers the interquartile range. The whiskers extend to 1.5 of the interquartile range and the cross *outside* is considered an outlier

The correlation between the predictions and experiments is fair as seen in Fig. 1a, with a correlation coefficient, *R* of 0.64, which is statistically significant (*p*-value < 0.001). For 77 % of the compounds the prediction of log *D* has the correct sign, and if we exclude predictions or experiments where log *D* is not significantly different from zero (determined by a *t*-test with a 95 % confidence level), the percentage of correctly predicted signs is 82 %. The correlation with experiment and percentage of correctly predicted signs are on a par with previously published predictions of water/hexane partition coefficients but slightly worse than predictions of water/octanol partition coefficients [23]. The deviations of the predictions range from 0.0 to 8.1 log units; the largest deviation is observed for compound **74**. This is also the only outlier in the error distribution as seen in the boxplot in Fig. 1b. The second largest deviation, 5.4 log units is observed for compound **75**. The mean absolute deviation (MAD) is 1.8 log units, which contains only a small systematic component, as the mean signed deviation (MSD) is only 0.3 log units. The root-mean-squared deviation (RMSD) is 2.4 log units. Compared to previous estimates of partition coefficients with the hybrid model [23], the MAD is significantly larger. For instance, hexane/water and octanol/water partition coefficients were predicted with MADs of 0.86 and 0.66 log units, respectively, i.e. about 1 log unit better than the cyclohexane log *D* values. There are of course many possible reasons for this, but two of the arguably most significant factors are the larger size of compounds in the SAMPL5 set and the fact that we are here trying reproduce experimental log *D* values rather than comparing to log *P* values as in the previous study. However we still compute log *P* values, and hence neglect the effects of tautomers and ionization.

To analyze the predictions further, we divided the compounds based on the chemical groups they contain. The objective is to see if compounds with specific moieties lead to significantly worse estimates than the other compounds. We used the checkmol program [35] to classify the compounds and could identify ten groups that contained at least five compounds and at most 47. All of them are listed in Table 2. The largest group is heterocyclic compounds, to which 47 compounds belong. The group of carboxylic acids and phenols only contains five compounds each. For these ten groups, we list in Table 2 the BEDROC metric, the *p*-value for a *t*-test of the absolute deviations for the group compared to the total population and the MSD. The analytical BEDROC value, assuming a uniform predictive power of all chemical groups, is listed as well, and serves as a yardstick to determine if the observed BEDROC value of a chemical group indicates a systematic deviation. We observe some BEDROC values that are larger than that expected from a uniform distribution, e.g. amines have an observed value of 0.65 compared to 0.50 for a uniform distribution. However, none of the differences between observed and uniform BEDROC value are significant at the 95 % confidence level, indicating that no particular chemical group is producing worse predictions than the other groups. This is also confirmed by the *p*-value of the absolute deviations that is larger than 0.05 for all groups; the smallest *p*-value is found for halogen derivatives, 0.08. Finally, the MSD for many groups is less than 1 log unit, also indicating a lack of systematic error. The largest MSDs are found for ethers, 1.9 log units and phenols, 1.8 log units. Thus, we can conclude that the deviations of the predictions compared to experiments are most likely random in nature.

**Table 2:** Analysis of the deviation between hybrid predictions and experiment for different chemical groups

Group	*N*	BEDROC			*p*-value	MSD
		Uniform	Observed			
Alcohol	8	0.44	0.57	±0.13	0.39	0.64
Amine	27	0.50	0.65	±0.08	0.30	0.74
Aromatic amine	13	0.46	0.45	±0.10	0.78	−0.92
Carboxylic acid	5	0.43	0.53	±0.09	0.79	−0.99
Carboxylic acid amide	18	0.47	0.35	±0.08	0.33	−0.03
Ether	17	0.47	0.54	±0.09	0.62	1.94
Halogen derivative	7	0.44	0.26	±0.08	0.08	0.62
Heterocyclic compound	47	0.56	0.24	±0.14	0.52	−0.07
Oxo(het)arene	6	0.44	0.18	±0.13	0.18	−0.80
Phenol	5	0.43	0.48	±0.09	0.93	1.78

Both the expected BEDROC value from a uniform distribution and the observed value are shown. The *p*-value is of a test of the unsigned deviation of the group compared to the entire population and MSD is the mean signed deviation

### Comparison with all-atom predictions

Arguably the main approximation of the submitted predictions lies in the simple CG model of the solvent molecules. Fortunately, we can make a rough quantification of the effect of this approximation by comparing to submissions that utilized all-atom solvents. There were several such submissions, but here we will only compare to a submission from the Mobley lab [37]. They used the same force field for the compounds and the same starting conformations as we used. There are some differences in the free energy methodology, but the length of the simulations is largely similar. Therefore, we consider this to be the closest all-atom submission to the hybrid AA/CG submission presented herein. The Mobley lab was also kind enough to provide the individual solvation free energies, which enables further analysis.

There are clear differences between the AA and AA/CG predictions as seen in Table 3. For Δ*G*_solv_ in water the absolute deviations range from 0.2 to 41.2 kJ/mol, with a MAD of 12.7 kJ/mol. The differences are systematic as the MAD is almost as large as the MSD, and in general the hybrid estimates of the hydration free energies are more negative than AA. The same holds true for the estimates in cyclohexane, but in this medium the deviations are smaller; the absolute deviations range from 0.8 to 13.5 kJ/mol with a MAD of 4.8 kJ/mol. For log *D* the deviations range from 0.1 to 6.2 log units, with a MAD of 1.7 log units. Thus it is clear that the deviations between the AA/CG and AA log *D* values are of similar magnitude as the deviations between the hybrid predictions and experiments (see Table 1). However, the correlation between the AA and hybrid predictions, *R* = 0.86 is stronger than the correlation between the hybrid predictions and experiment, *R* = 0.64. In fact, the correlation between an AA and AA/CG is stronger for the estimates of Δ*G*_solv_, but because the slope is different in the two media this correlation does not translate to log *D*.

**Table 3:** Statistics on the deviation between hybrid and all-atom estimates

	Δ*G*_solv_ (water)	Δ*G*_solv_ (cyclohexane)	log *D*
MAD	12.7	4.8	1.7
MSD	12.2	4.8	1.3
MAX^a^	41.2	13.2	6.2
*R*	0.94	1.00	0.86
Slope	0.80	0.92	0.76

Solvation free energies in kJ/mol

^a^MAX is the maximum absolute deviation

The predictions of Δ*G*_solv_ for compound **74** differ by 41.2 and 5.6 kJ/mol in water and cyclohexane, respectively. Thus, it is clear that the difference between the AA and AA/CG models is manifested differently in the two media. We investigated this further by computing the BEDROC metric of the same ten groups used above, but here we analyze the difference between the AA and AA/CG estimates of Δ*G*_solv_ and log *D*. For the predictions of Δ*G*_solv_ in water, we observe a BEDROC metric that is significantly larger than expected from a uniform distribution for aromatic amines, carboxylic acids, heterocyclic compounds and phenols (see Table 4). For all of these groups, except phenols, the significantly larger BEDROC values are also observed with log *D*. For the predictions in cyclohexane, we only observe significantly larger BEDROC values for amines and ethers, which is not translated to the log *D* estimates. Thus, we see that compounds with some chemical groups give large differences in water, and compounds with other groups give large differences in cyclohexane. Whether these differences also give large differences in log *D* depends on the individual compounds. It is also striking that there is no apparent trend among the groups that show large differences. For instance, it is not immediately clear why we observe a significantly larger BEDROC value for aromatic amines in water, but not for all amines, whereas the opposite is true in cyclohexane.

**Table 4:** BEDROC metric of the deviation between hybrid and all-atom predictions for different chemical groups

Group	Δ*G*_solv_ (water)		Δ*G*_solv_ (cyclohexane)		log *D*	
Alcohol	0.48	±0.11	0.69	±0.13	0.34	±0.12
Amine	0.58	±0.08	**0.80**	**±0.06**	0.56	±0.08
Aromatic amine	**0.76**	**±0.08**	0.41	±0.09	**0.76**	**±0.08**
Carboxylic acid	**0.70**	**±0.07**	0.23	±0.06	**0.74**	**±0.07**
Carboxylic acid amide	0.39	±0.08	0.52	±0.09	0.38	±0.09
Ether	0.42	±0.09	**0.76**	**±0.08**	0.33	±0.08
Halogen derivative	0.36	±0.08	0.15	±0.11	0.43	±0.11
Heterocyclic compound	**0.86**	**±0.06**	0.33	±0.10	**0.91**	**±0.04**
Oxo(het)arene	0.44	±0.17	0.48	±0.13	0.47	±0.17
Phenol	**0.83**	**±0.07**	0.69	±0.13	0.69	±0.10

The observed values that are significantly larger than BEDROC metrics for a uniform distribution (see Table 2) are shown in bold

## Conclusion

We have presented a submission to the SAMPL5 challenge on distribution coefficients. Our methodology is simple and efficient: we approximate the distribution coefficient by the partition coefficient through the estimation of solvation free energies in water and cyclohexane, employing a hybrid all-atom/coarse-grained model. Such an approach is at least ten times faster than a corresponding all-atom approach [21, 22]; a solvation free energy in water and cyclohexane is computed in 13 and 7 CPU hours on average, respectively on 12 cores of a Cray XC30 machine. We have previously used this hybrid model to produce hexane/water and octanol/water predictions with high accuracy both in comparison to experiment and to a more expensive all-atom solvent model [23]. The SAMPL5 predictions presented herein are a further testament to the accuracy and robustness of this computationally inexpensive model. We obtain a mean absolute deviation of 1.8 log units and a significant correlation coefficient, *R* of 0.64. In addition, 84 % of the predictions had the correct sign, which is arguably the most important quality for a model predicting partitioning. The estimates seem to be without any systematic bias, and neither is the model more sensitive to a particular chemical group. This observed quality of the AA/CG predictions is on a par with or better than the other submissions employing a simulation approach with a fixed-charged atomistic force field [37]. However, the deviations to experiments are larger than what was expected from previous estimates of log *P* [23] and there are several possible reasons for this: The compounds in the SAMPL5 challenge are larger, which is also seen in the increased uncertainty of the estimates. Furthermore, we compare to experimental log *D*, and hence neglect the contribution from all but one tautomer and the possible ionization in the water phase. The much better quality of cyclohexane/water log *P* values for 79 compounds from the Minnesota database [38] presented in the Supplementary Material, is a clear indication of this. Thus, it seems that the logical place to start on improvements is to add corrections to the log *P* estimates accounting for different tautomers and ionization effects. However, such corrections are far from accurate or complete [37], and therefore we argue that corrections have to be the subject of future investigations. Other possible error sources include the neglect of a finite water concentration in the cyclohexane phase, compound dimerization, and experimental setup. Even so, the results herein clearly show that a majority of the physics involved in the partitioning of small molecules between water and cyclohexane is captured with a simple CG solvent model.

## Electronic supplementary material

Below is the link to the electronic supplementary material.

Supplementary material 1 (DOCX 122 kb)

Supplementary material 2 (XLSX 61 kb)
