# Supplementary material for: All-atom/coarse-grained hybrid predictions of distribution coefficients in SAMPL5
Source: J Comput Aided Mol Des. 2016 Jul 26;30(11):969–76. doi: 10.1007/s10822-016-9926-z (PMC5206257; doi:10.1007/s10822-016-9926-z)
Supplement: Supplementary file 1 — Supplementary material 1 (DOCX 122 kb) [file 10822_2016_9926_MOESM1_ESM.docx]

Supplementary Material:

All-atom/coarse-grained hybrid predictions of distribution coefficients in SAMPL5

Samuel Genheden^1*^, Jonathan W. Essex^2^

^1^ Department of Chemistry and Molecular Biology, University of Gothenburg, Box 462, SE-405 30, Göteborg, Sweden

^2^School of Chemistry, University of Southampton, Southampton, UK, SO17 1BJ

* Correspondence to samuel.genheden@gu.se

**Validation of coarse-grained cyclohexane model**

The coarse-grained (CG) models of water and cyclohexane are shown in Figure S1. 512 cyclohexane molecules were placed in a box with the size set according to the experimental density. The system was equilibrated for 100 ps in the NVT ensemble and for 100 ps in the NPT ensemble, followed by a 10 ns production run in the NPT ensemble. All simulations were performed with the Lammps software [^[[1]](#endnote-1)^], employing a 10 fs timestep. A Langevin thermostat [^[[2]](#endnote-2)^] was used, fixing the temperature at 298 K with 10 ps time constant. The pressure was kept at 1 atm with a weak-coupling algorithm [^[[3]](#endnote-3)^], employing a 1 ps time constant. The density, *ρ,* was computed from the instantaneous volume and the enthalpy of vaporization, Δ*H*_vap_, was calculated using established protocols [^[[4]](#endnote-4)^]. Without applying a scaling factor to the Lennard-Jones parameters (as discussed in the main text) the CG model has a density of 542 g/L compared to an experimental value of 778 g/L [^[[5]](#endnote-5)^], i.e. too low. However, with the applied scaling, the CG model has a density of 668.5 g/L, i.e. much closer to the experimental value. The relative error is only 14%, and comparable to the CG model of hexane [^[[6]](#endnote-6)^]. The experimental Δ*H*_vap_ of 33.1 kJ/mol [5] is overestimated in the CG model, which gave an average Δ*H*_vap_ = 54.8 kJ/mol. This is only much worse than for the CG model of hexane, which only showed a discrepancy of 2 kJ/mol compared to experiment [6].


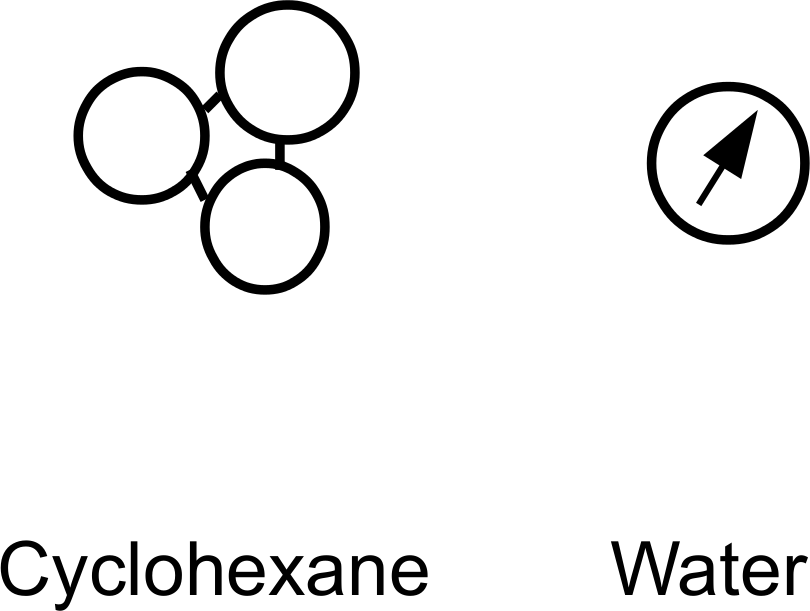


**Figure S1 ­–** Representation of the CG models used herein. Each sphere represents a Lennard-Jones particle and the arrows indicate point dipoles.

To validate the ability of the CG model to estimate log *P*, we computed solvation free energies of 79 compounds in the Minnesota Solvation database [^[[7]](#endnote-7)^]. These compounds were selected among the compounds for which we have previously estimated hydration free energies [6] and that also have an experimentally determined solvation free energy in cyclohexane. The compounds were solvated in cyclohexane as described previously [6]. The solvation free energies followed the same protocol as described for the Sampl5 challenge in the main text, except that only 2.4 ns sampling was performed at each value of *λ* with 600 ps discarded as equilibration. Furthermore, only two independent repeats were used. All of the estimates are collected in the Supplementary Material spreadsheet. The MAD of the log *P* calculations is 0.89 log units, comparable to the estimates of hexane/water and octanol/water partition coefficients [6]. The correlation coefficient is excellent as seen in Figure S2; *R* = 0.86 and statistically significant. Thus we can conclude that our simple CG model of cyclohexane, despite not reproducing all properties of the pure liquid, can produce accurate estimates of partition coefficients.


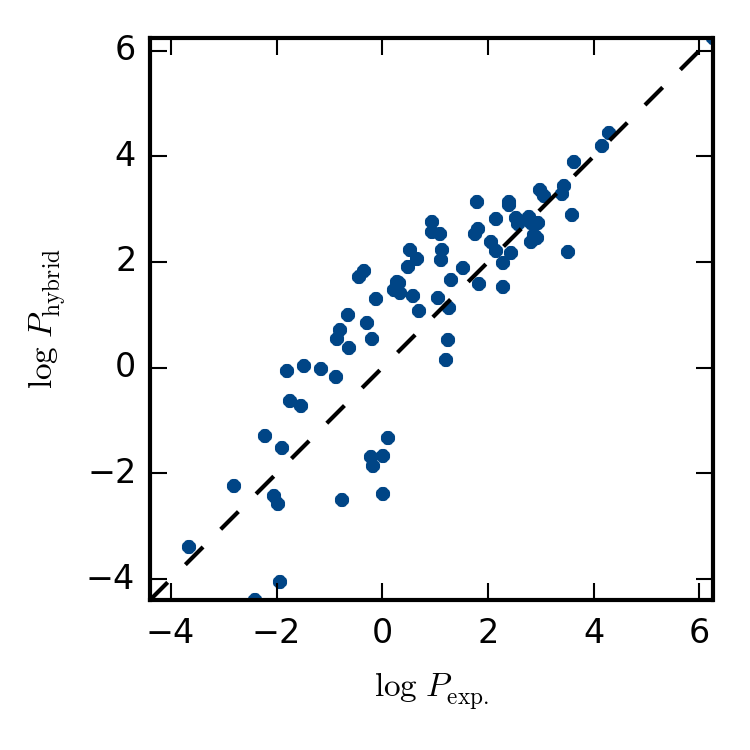


**Figure S2** – Correlation between experimental and predicted cyclohexane/water log *P* for 79 compounds in the Minnesota solvation database

**References**

1. Plimpton S (1995) Fast Parallel Algorithms for Short-Range Molecular Dynamics. J Comput Phys 117:1–19. doi: 10.1006/jcph.1995.1039 [↑](#endnote-ref-1)
2. Hünenberger PH (2005) Thermostat algorithms for molecular dynamics simulations. Adv Polym Sci 173:105–147. doi: 10.1007/b99427 [↑](#endnote-ref-2)
3. Berendsen HJC, Postma JPM, van Gunsteren WF, et al (1984) Molecular dynamics with coupling to an external bath. J Chem Phys 81:3684. [↑](#endnote-ref-3)
4. Orsi M (2013) Comparative assessment of the ELBA coarse-grained model for water. Mol Phys 112:1–11. doi: 10.1080/00268976.2013.844373 [↑](#endnote-ref-4)
5. Lide DR (2004) CRC Handbook of Chemistry and Physics 85th edition. CRC Press. Cleveland, Ohio [↑](#endnote-ref-5)
6. Genheden S (2016) Predicting Partition Coefficients with a Simple All-Atom/Coarse-Grained Hybrid Model. J Chem Theory Comput 12:297–304. doi: 10.1021/acs.jctc.5b00963 [↑](#endnote-ref-6)
7. Marenich AV, Kelly CP, Thompson JD, Hawkins GD, Chambers, CC, Giesen DJ, Winget P, Cramer CJ, Truhlar DG (2012) Minnesota Solvation Database – version 2012. University of Minnesota, Minneapolis. [↑](#endnote-ref-7)
